# Overexpression of FoxM1 Enhanced the Protective Effect of Bone Marrow-Derived Mesenchymal Stem Cells on Lipopolysaccharide-Induced Acute Lung Injury through the Activation of Wnt/*β*-Catenin Signaling

**DOI:** 10.1155/2023/8324504

**Published:** 2023-02-11

**Authors:** Yuling Luo, Shan Lin, Xueyan Mao, Yongqiang Yang, Wanmei He, Manliang Guo, Mian Zeng

**Affiliations:** ^1^Department of Medical Intensive Care Unit, The First Affiliated Hospital, Sun Yat-sen University, Guangzhou, Guangdong, China; ^2^Department of Respiratory and Critical Care Medicine, Affiliated Hospital of North Sichuan Medical College, Nanchong, Sichuan, China

## Abstract

**Background:**

Mesenchymal stem cell- (MSC-) based cell and gene therapies have made remarkable progress in alleviating acute lung injury/acute respiratory distress syndrome (ALI/ARDS). However, the benefits of Forkhead box protein M1 (*FoxM1*) gene-modified MSCs in the treatment of ALI have not been studied.

**Methods:**

We evaluated the therapeutic effects of *FoxM1*-modified MSCs in ALI mice induced by lipopolysaccharide (LPS) by quantifying the survival rate, lung weight ratio (wet/dry), and contents of bronchoalveolar lavage fluid. In addition, microcomputed tomography, histopathology, Evans Blue assay, and quantification of apoptosis were performed. We also explored the underlying mechanism by assessing Wnt/*β*-catenin signaling following the treatment of mice with *FoxM1*-modified MSCs utilizing the Wnt/*β*-catenin inhibitor XAV-939.

**Results:**

Compared with unmodified MSCs, transplantation of *FoxM1*-modified MSCs improved survival and vascular permeability; reduced total cell counts, leukocyte counts, total protein concentrations, and inflammatory cytokines in BALF; attenuated lung pathological impairments and fibrosis; and inhibited apoptosis in LPS-induced ALI/ARDS mice. Furthermore, *FoxM1*-modified MSCs maintained vascular integrity during ALI/ARDS by upregulating Wnt/*β*-catenin signaling, which was partly reversed via a pathway inhibitor.

**Conclusion:**

Overexpression of FoxM1 optimizes the treatment action of MSCs on ALI/ARDS by inhibiting inflammation and apoptosis and restoring vascular integrity partially through Wnt/*β*-catenin signaling pathway stimulation.

## 1. Introduction

Acute lung injury/acute respiratory distress syndrome (ALI/ARDS) is distinguished by increased lung permeability, pulmonary edema, and infiltration of inflammatory cells [1], with mortality rates ranging from 35% to 46% [2]. Damage of alveolar epithelial-endothelial barriers is an important aspect of ARDS pathophysiology [3, 4]. Currently, no specific drug therapy is effective for patients with ARDS, and all available treatments involve supportive measures [2]. Therefore, further investigation of promising therapeutic strategies that target these pathophysiological features of ALI/ARDS is urgently needed.

Transplantation of mesenchymal stem cells (MSCs), a multifunctional cell type, is considered a promising approach for the treatment of ARDS due to their immunomodulatory and anti-inflammatory properties [5, 6]. Administration of MSCs reduces alveolar-capillary barrier damage and mortality in preclinical models of ALI [7–11]. Although the mechanism by which MSCs exert benefit is unclear, increasing evidence suggests that it is related to their secretory functions. Indeed, through paracrine effects, MSCs regulate important pathologic pathways of ARDS and sepsis such as inflammation, damage of endothelial and epithelial cells, clearance of alveolar fluid, antimicrobial action, and apoptosis [12]. Several paracrine mediators of MSCs have been identified, including interleukin 10 (IL-10) [13], antimicrobial peptide lipocalin 2 [14], angiopoietin 1 [8, 15, 16], keratinocyte growth factor [17, 18], hepatocyte growth factor [19], and the antimicrobial peptide LL-37 [20]. Moreover, recent evidence indicates that gene-modified MSCs can further enhance their effects in the treatment of ALI [21].

Forkhead box (FOX) proteins, such as Forkhead box protein M1 (FoxM1), represent a broad family of transcriptional regulators that regulate cell cycle progression, proliferation, differentiation, metabolism, aging, survival, and apoptosis [22]. FoxM1 activity ensures proper epithelial and mesenchymal tissue development during embryonic and fetal development [23] and takes part in repairing organ damage like the lung, liver, heart, and spinal cord during disease [24–27]. In addition, we found that the therapeutic efficacy of bone marrow-derived MSCs (BMSCs) was enhanced by overexpression of FoxM1 in preliminary experiments evaluating lipopolysaccharide- (LPS-) induced endothelial cell (EC) injury. Thus, FoxM1 genetically engineered MSCs are a possible treatment choice for ALI. Moreover, whether FoxM1 enhances the therapeutic effect of MSCs on ALI/ARDS in vivo is still uncertain. Therefore, the present research sought to examine the pathway by which BMSCs overexpressing FoxM1 alleviate ALI/ARDS.

## 2. Materials and Methods

### 2.1. BMSC Transduction and Culture

Lentiviral transfection of Sprague-Dawley rat BMSCs was performed as previously reported [21]. Successfully transfected cells were grown in Dulbecco's modified Eagle medium mixed with 10% fetal bovine serum as well as 1% streptomycin/penicillin in a carbon dioxide incubator. Three to ten passages of cells were employed in further tests.

### 2.2. Preparing Experimental Animals

Sun Yat-sen University (Guangzhou, China) supplied all animals. Each procedure was authorized by the Animal Care and Use Committee of the First Hospital of Sun Yat-sen University. To establish the ALI mouse model, mice were given LPS (10 mg/kg, Escherichia coli 055:B5; Sigma-Aldrich; USA) via injection intraperitoneally [28, 29]. Mice were originally split into four groups (12 mice per group): control, LPS, LPS+BMSCs-Vector, and LPS+BMSCs-FoxM1. After 4 h of LPS exposure, BMSCs-Vector or BMSCs-FoxM1 (1 × 10^6^ cells/200 *μ*L saline) were supplemented by injection into the mice's tail veins in the respective groups, whereas LPS and control group mice received 200 *μ*L of saline. Twenty-four hours later, mice were sacrificed, and their lung tissues were collected for follow-up experiments.

In addition, we explored the underlying mechanism by assessing Wnt/*β*-catenin signaling following the treatment of mice with *FoxM1*-modified BMSCs utilizing the Wnt/*β*-catenin suppressor XAV-939 (APExBIO, USA).

### 2.3. Lung Wet/Dry Weight Ratio

The right upper lungs of each animal were extracted and weighed instantly to assess the wet weight. To assess the dry weight, lung tissues were dehydrated for 48 h in a drying oven at 60°C. Then, the lung wet/dry ratio (*W*/*D*) was detected as follows: *W*/*D* = dry weight/wet weight × 100%.

### 2.4. Evans Blue (EB) Assay

To evaluate pulmonary barrier permeability, EB dye (Sigma-Aldrich) was given through injecting the tail vein (20 mg/kg) of each mouse 1 h before sacrifice [30]. Subsequently, mice were injected intracardially with sterile saline, and their lungs were rapidly removed. After weighing, lung tissues were homogenized in N-dimethylformamide, incubated for 72 h at 60°C, and then centrifuged at 5000 × g for 30 min to isolate EB dye. Finally, the EB dye absorption was determined at 630 nm with a spectrophotometer.

### 2.5. Histology

Lung tissue samples were fixed in paraffin and had hematoxylin and eosin (H&E) as well as Masson's trichrome staining. Finally, Smith [31] and Ashcroft [32] scores were used to evaluate pathological changes and the degree of pulmonary fibrosis, respectively.

### 2.6. Microcomputed Tomography (Micro-CT) Experiment

At 24 h post-LPS exposure, lung images were acquired by a micro-CT device (Bruker, Billerica, MA) to assess the extent of lung infiltration.

### 2.7. Immunofluorescence Staining

Lung sections were incubated overnight at 4°C with an antibody against vascular endothelial- (VE-) cadherin (Abcam, Cambridge, UK) and *β*-catenin (Cell Signaling Technology, Danvers, MA), rinsed thrice with PBS (10 min each), and then incubated for 1 h with second antibodies at room temperature in darkness. Afterwards, sections were incubated for 10 min with DAPI at room temperature. Lastly, a fluorescence microscope (Olympus, Tokyo, Japan) was utilized to image tissue samples.

### 2.8. Evaluation of Leukocyte Influx and Protein Level in Bronchoalveolar Lavage Fluid (BALF)

BALF was collected in accordance with a previously published protocol [33]. Total cells in BALF were detected with a hemocytometer, and total protein was evaluated with a bicinchoninic acid (BCA) assay kit (Beyotime, Haimen, China). Additionally, numbers of leukocytes were counted after staining with Wright-Giemsa dye.

### 2.9. Enzyme-Linked Immunosorbent Assay (ELISA)

Corresponding ELISA kits (eLGbio, Guangzhou, China) were utilized to recognize contents of tumor necrosis factor *α* (TNF-*α*) as well as IL-1*β*, IL-6, IL-4, and IL-10 inflammatory factors in BALF.

### 2.10. Western Blot

Total proteins were isolated utilizing RIPA buffer with 1% protease inhibitor and then quantified utilizing the BCA assay. After loading and electrophoresis of equal amounts of protein in 10% sodium dodecyl sulfate-polyacrylamide gels, the proteins were subsequently transmitted onto polyvinylidene fluoride membranes. The membranes were blocked with 5% dry skimmed milk for 1 h at room temperature and after were incubated at 4°C overnight with the next primary antibodies: anti-*β*-actin (DEWEIBIO, Guangdong, China), anti-*β*-catenin, anti-VE-cadherin, anti-Bcl-2, and anti-Bax. Following three washes with Tris-buffered saline comprising Tween 20, blots with the respective enzyme-linked secondary antibody were incubated for 1 h at room temperature. Next, protein bands were determined by improved chemiluminescence staining utilizing a kit (Merck Millipore, USA) and ImageJ software v1.4.0 (http://imagej.nih.gov), with *β*-actin as the loading control.

### 2.11. Statistical Methods

Measurement data were displayed as the mean ± SEM. One-way ANOVA and Tukey's test were utilized to compare multiple groups, whereas two groups were compared utilizing Student's *t*-test. *p* values below 0.05 were assessed as statistically significant.

## 3. Results

### 3.1. Injection of BMSCs Overexpressing FoxM1 Enhanced Survival and Ameliorated LPS-Induced ALI Mice

**Figure 1:**
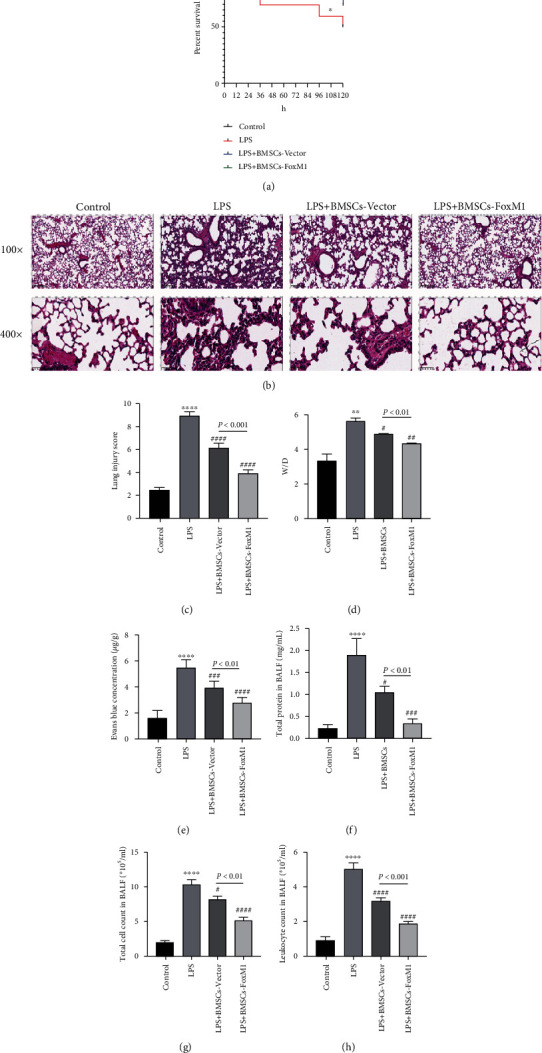
BMSCs-FoxM1 alleviate LPS-induced ALI mice: (a) Kaplan-Meier survival curves (*n* = 10); (b, c) severity and score of lung injury, magnification 100x and 400x (*n* = 6); (d) lung wet/dry assay (*n* = 6); (e) Evans Blue dye evaluates pulmonary barrier permeability (*n* = 6). The detection of total protein concentration (f), total cell count (g), and leukocyte count (h) in BALF (*n* = 6). Values are reported as the mean ± SEM. ^∗^*p* < 0.05,  ^∗∗^*p* < 0.01,  ^∗∗∗∗^*p* < 0.0001 whereas for control group; ^#^*p* < 0.05, ^##^*p* < 0.01, ^###^*p* < 0.001, ^####^*p* < 0.0001 vs. LPS group.

For the survival study, the survival of mice was monitored after LPS exposure. At the end of the 5-day follow-up period, the survival rate of mice in the control group was 100%, whereas it was 50% in the LPS group. Remarkably, the survival rate of mice in the LPS+BMSCs-Vector and LPS+BMSCs-FoxM1 groups was significantly increased, especially in the LPS+BMSCs-FoxM1 group (Figure 1(a)). To evaluate pathological changes in the lung, paraffin sections were stained with H&E. Lung histopathology revealed that lung injuries include inflammatory cell infiltration, thickened alveolar walls, and edema with hemorrhaging edema in LPS-treated mice, whereas these pathological changes were significantly attenuated in LPS-induced ALI mice treated with BMSCs-Vector or BMSCs-FoxM1, especially BMSCs-FoxM1 posttreatment (Figure 1(b)). These pathological changes were reflected in the lung injury score (Figure 1(c)). These results indicated that overexpression of FoxM1 could enhance BMSCs improving survival and alleviating lung tissue damage of LPS-induced ALI mice.

One of the typical pathological features of ALI is dysfunction of the pulmonary vascular endothelial barrier. To investigate the effects of *FoxM1*-modified BMSCs on pulmonary vascular permeability, we quantified *W*/*D* weight ratios, extravasation (as evaluated with Evans Blue dye), and BALF protein concentrations. The findings showed that *W*/*D* weight ratios in the LPS+BMSCs-FoxM1 group were significantly decreased compared with the BMSCs-Vector and LPS groups (Figure 1(d)). Similarly, protein concentrations in BALF and quantitative extravasation of Evans Blue dye, as well as *W*/*D*, were remarkably reduced in mice treated with FoxM1-modified BMSCs (Figures 1(e) and 1(f)). These findings illustrated that *FoxM1*-modified BMSCs had a protective effect against pulmonary vascular permeability in LPS-induced ALI mice. These results indicated that overexpression of FoxM1 could enhance BMSCs improving the vascular permeability of LPS-induced ALI mice.

**Figure 2:**
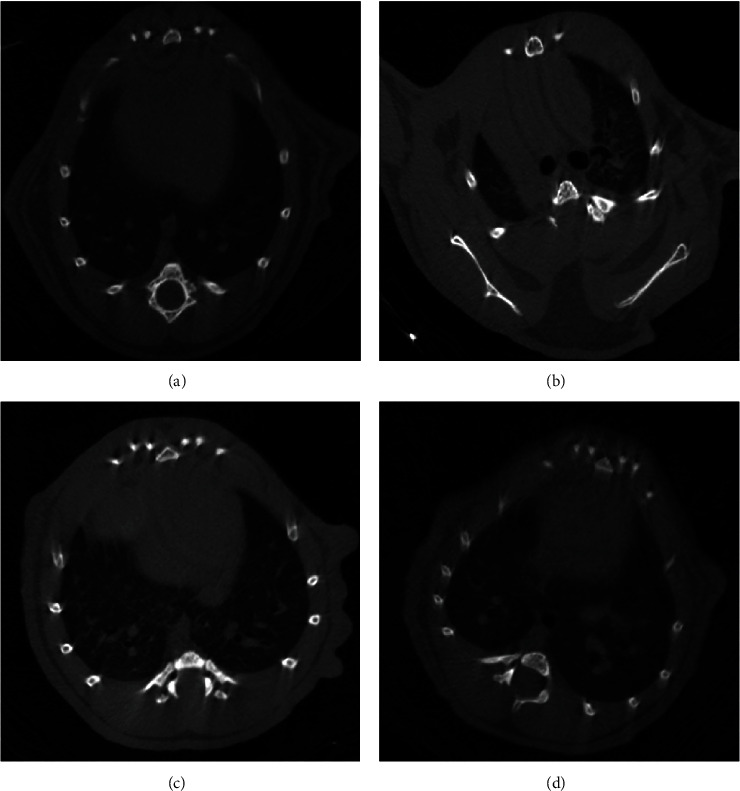
Extent of lung infiltration assessed by microcomputed tomography: (a) control group; (b) LPS group; (c) LPS+BMSCs-Vector group; (d) LPS+BMSCs-FoxM1.

The number of neutrophils in BALF is an indicator of the degree of inflammatory damage in lung tissue. As shown in Figures 1(g) and 1(h), strong inflammation was confirmed in LPS group mice by significantly increased counts of total cells and leukocytes in BALF, which were significantly lowered in mice receiving either BMSCs treatment, particularly BMSCs-FoxM1. Additionally, micro-CT revealed bilateral patchy infiltrates in LPS group mice compared with the normal radiological aspects of the lung in controls; however, these patchy infiltrates were significantly decreased in the LPS+FoxM1 group contrasted to the LPS and LPS+BMSCs-Vector groups (Figure 2). Overall, these results confirm that overexpression of FoxM1 enhanced the inflammation inhibition effect of BMSCs on LPS-induced lung injury.

### 3.2. BMSCs Overexpressing FoxM1 Alleviated Pulmonary Fibrosis Induced by LPS

**Figure 3:**
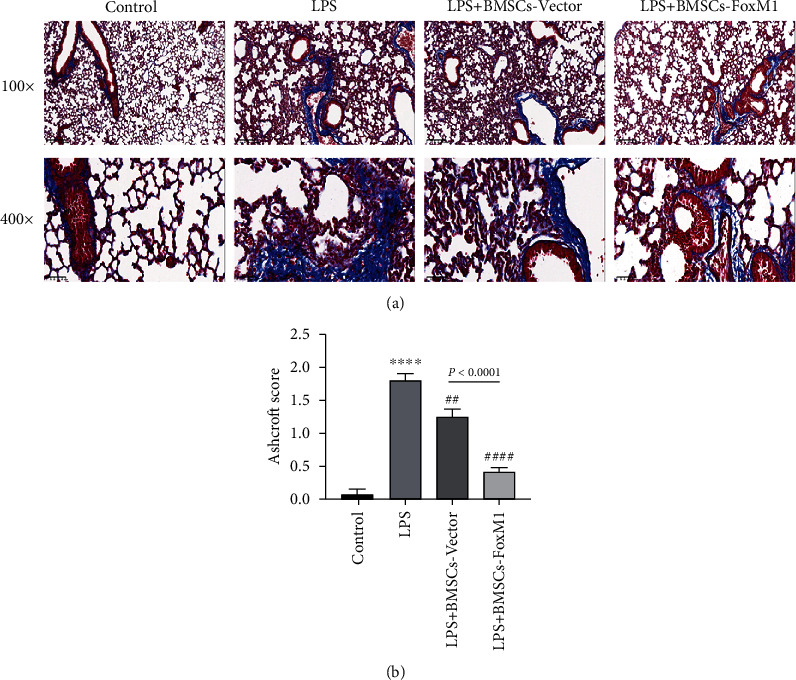
BMSCs-FoxM1 alleviated pulmonary fibrosis of LPS-induced ALI: (a) Masson staining lung sections; (b) grading of lung fibrosis. Values are provided as the mean ± SEM. *n* = 6, ^∗∗∗∗^*p* < 0.0001 whereas for control group; ^##^*p* < 0.01, ^####^*p* < 0.0001 vs. LPS group.

Studies have shown that fibrosis may occur in the early stage of ARDS [34, 35]. BMSC treatment has been shown to markedly reduce the collagen deposition observed in LPS-induced lung injury [36]. Therefore, Masson staining was used to evaluate the effect of FoxM1-modified BMSCs on ALI fibrosis. The images of Masson staining revealed severe collagen deposition and fibrotic lesions in LPS group mice contrasted to the control group; however, less damage was discovered in the LPS+BMSCs-FoxM1 group contrasted to the LPS and LPS+BMSCs-Vector groups (Figure 3(a)). We next used the Ashcroft score to quantify lung fibrosis in each group. The results showed that the Ashcroft score in the LPS+BMSCs-FoxM1 group was significantly decreased compared with the BMSCs-Vector and LPS groups (Figure 3(b)). These findings indicated that overexpression of FoxM1 could enhance the fibrosis inhibition effect of BMSCs on LPS-induced lung injury.

### 3.3. Measuring Inflammatory Factors

**Figure 4:**
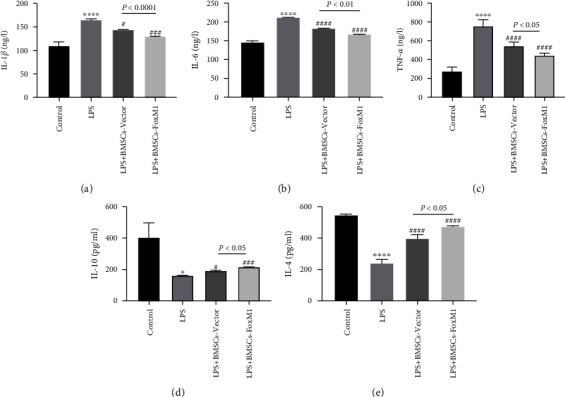
BMSCs-FoxM1 regulates LPS-induced inflammation in BALF. BMSCs-FoxM1 reduced LPS-induced protein expression of IL-1*β* (a), IL-6 (b), and TNF-*α* (c). BMSCs-FoxM1 suppressed the decrease of LPS-induced production of the pro-anti-inflammatory IL-10 (d) and IL-4 (e). Values are reported as the mean ± SEM. *n* = 3-6, ^∗^*p* < 0.05,  ^∗∗∗∗^*p* < 0.0001 whereas for control group; ^#^*p* < 0.05, ^###^*p* < 0.001, ^####^*p* < 0.0001 vs. LPS group.

ALI is a complex inflammatory disease, and inflammatory cytokine release has been proven to perform a pivotal function in the pathologic process of sepsis-stimulated lung injury. Therefore, we detected inflammatory cytokines using ELISA. ELISA detected levels of pulmonary inflammatory cytokines (Figure 4). IL-1*β*, IL-6, and TNF-*α* levels in BALF were significantly raised in the LPS group in contrast to the control group, while IL-4 and IL-10 were significantly decreased. The BMSCs-FoxM1 group exhibited lower levels of inflammatory factors (IL-1*β*, IL-6, and TNF-*α*) and higher levels of anti-inflammatory factors (IL-4 and IL-10) compared with the LPS and LPS+BMSCs-Vector groups. These findings indicated that overexpression of FoxM1 could enhance the lung inflammation inhibition effect of BMSCs on LPS-induced lung injury.

### 3.4. BMSCs Overexpressing FoxM1 Activated the Wnt/*β*-Catenin Signaling Pathway In Vivo

**Figure 5:**
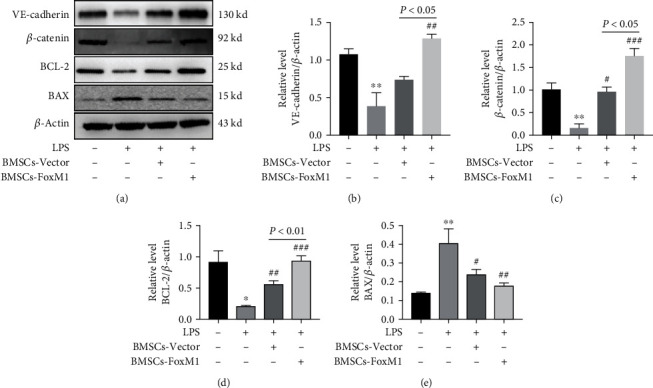
BMSCs-FoxM1 protects against LPS-induced ALI via stimulating the Wnt/*β*-catenin pathway: (a) Western blotting evaluated the expression of Wnt/*β*-catenin, VE-cadherin, and apoptosis-related proteins (Bcl-2, Bax); (b–e) Western blot densitometric evaluation. Values are presented as the mean ± SEM. *n* = 3, ^∗^*p* < 0.05,  ^∗∗^*p* < 0.01 whereas for control group; ^#^*p* < 0.05, ^##^*p* < 0.01, ^###^*p* < 0.001 vs. LPS group.

**Figure 6:**
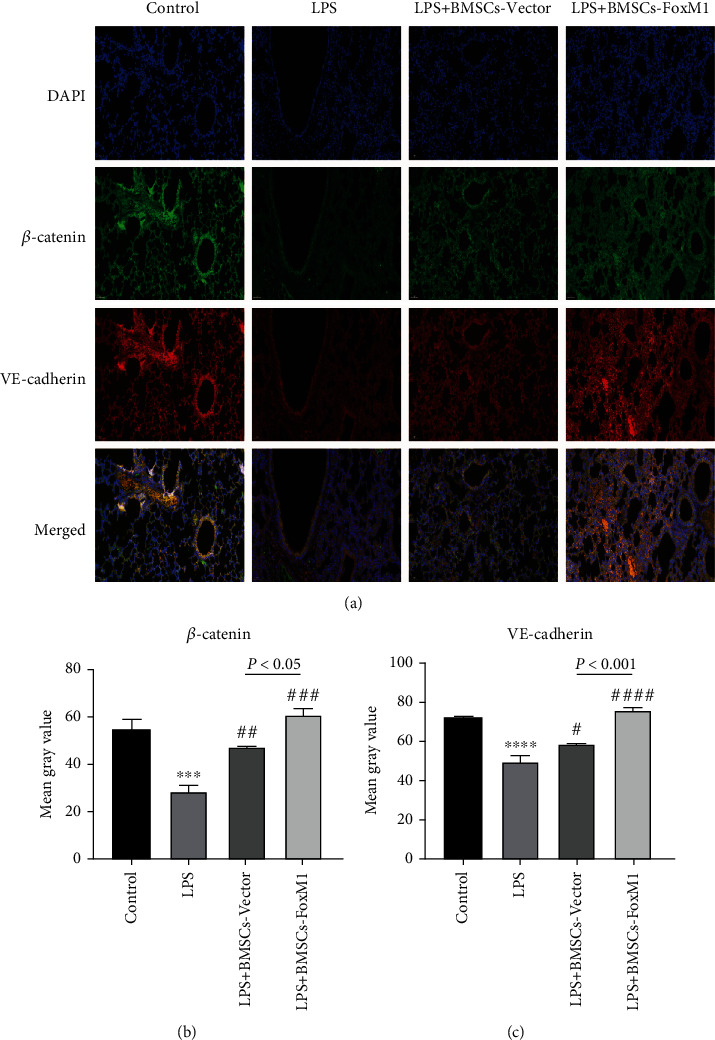
Impact of BMSCs-FoxM1 on *β*-catenin and VE-cadherin expression in LPS-induced ALI mice by immunofluorescence analysis: (a) immunofluorescence staining for *β*-catenin (green) and VE-cadherin (red); (b) quantitative *β*-catenin expression; (c) quantitative VE-cadherin expression. Values are reported as the mean ± SEM. *n* = 3,  ^∗∗∗^*p* < 0.001, ^∗∗∗∗^*p* < 0.0001 whereas for control group; ^#^*p* < 0.05, ^##^*p* < 0.01, ^###^*p* < 0.001, ^####^*p* < 0.0001 vs. LPS group.

**Figure 7:**
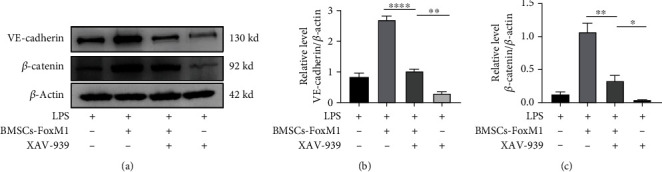
The effect of Wnt/*β*-catenin pathway inhibitor (XAV-939) on BMSCs-FoxM1 in the treatment of LPS-induced ALI: (a) the expression of VE-cadherin and *β*-catenin; (b, c) Western blot densitometric evaluation. Values are reported as the mean ± SEM. *n* = 3,  ^∗^*p* < 0.05,  ^∗∗^*p* < 0.01,  ^∗∗∗∗^*p* < 0.0001.

VE-cadherin, a key member of adherens junctions [37], plays a vital part in controlling pulmonary microvascular endothelial permeability. To study the protective pathway of FoxM1-modified BMSCs on endothelial barrier integrity, we examined VE-cadherin, *β*-catenin, Bcl-2, and Bax protein expressions. We observed that protein levels of VE-cadherin, *β*-catenin, and the antiapoptotic protein Bcl-2 were significantly reduced, while apoptosis-associated protein Bax was significantly raised in the LPS group contrasted to the control group (Figures 5(a)–5(e)). Moreover, *β*-catenin, VE-cadherin, and the antiapoptotic protein Bcl-2 expressions were significantly elevated, and apoptosis-associated protein Bax expression was significantly reduced in the LPS+BMSCs-FoxM1 group contrasted to the LPS and LPS+BMSCs-Vector groups (Figures 5(a)–5(e)). Similarly, immunofluorescence results show significantly decreased expression of VE-cadherin and *β*-catenin in the LPS group, while these factors were markedly increased in the LPS+BMSCs-FoxM1 group (Figures 6(a)–6(c)). Intriguingly, a particular Wnt/*β*-catenin pathway suppressor (XAV-939) abolished the effect of BMSCs-FoxM1, which downregulated not only protein expression of *β*-catenin but also VE-cadherin (Figures 7(a)–7(c)). Taken together, these results indicated that BMSCs overexpressing FoxM1 enhanced the protective effect of BMSCs on LPS-induced ALI through the activation of Wnt/*β*-catenin signaling.

## 4. Discussion

Herein, we examined the impacts of FoxM1-modified BMSCs on LPS-induced ALI. FoxM1 overexpression significantly stimulated the protecting impacts of BMSCs on LPS-induced ALI, partly by stimulating the Wnt/*β*-catenin signaling pathway. This research presents unique perspectives on MSC-based therapeutic strategies for ALI/ARDS.

MSCs have substantial treatment promises for ALI/ARDS. However, the potential mechanisms involved remain unclear. Recent preclinical studies have demonstrated that MSCs influence essential pathobiological mechanisms in ARDS and sepsis by releasing paracrine factors [12], which exert anti-inflammatory [38, 39] and antiapoptotic [21] effects and reduce the permeability of alveolar epithelium [40] and vascular endothelium [41]. Modifying certain genes can further induce the impact of MSCs in treating ALI [42–44]. Salerno et al. [45] found that aging of MSCs *in vitro* leads to the loss of chondrogenesis, and reduced chondrogenesis is associated with the downregulation of FoxM1 signaling. Xu et al. [46] found that activation of the ERK/FoxM1 pathway exerts protective effects against MSC senescence. In our study, BMSCs were modified with a retrovirus carrying *FoxM1* and then intravenously infused into mice 4 h after LPS-induced ALI modeling. Consistent with previous studies, we found that BMSCs-Vector reduced mortality, pulmonary edema, pathological damage, inflammatory cell recruitment into the lungs, fibrosis, and proinflammatory cytokine levels. As expected, *FoxM1*-modified BMSCs were more effective than BMSCs-Vector at producing therapeutic effects in a mouse model of ALI/ARDS.

ALI/ARDS pathogenesis involves alveolar capillary damage, which increases vascular permeability [47]. The importance of maintaining pulmonary endothelial integrality in the treatment of ARDS has been demonstrated [48]. In addition, some studies identified critical roles for FoxM1 in vascular repair [24, 49–53]. For example, Zhao et al. found that endothelial expression of FoxM1 is crucial for protecting bone marrow progenitor cells from LPS-induced lung inflammation and death [24]. In a mouse model with EC-restricted disruption of FoxM1, Mirza et al. demonstrated that FoxM1 repairs endothelial adherens junctions via *β*-catenin transcriptional control [51]. Moreover, abnormally activating FoxM1 can lead to overexpression of MMP-2, VEGF, ZEB1, and so on, which are all angiogenic genes [54]. Here, consistent with previous findings, we demonstrated that FoxM1 enhances BMSCs to restore vascular integrity by reducing vascular leakage and exerting antiapoptotic impacts (lowering Bax and elevating Bcl-2 activity). VE-cadherin, a key member of adherens junctions, regulates endothelial permeability in vessels [55]. Interestingly, we observed that *FoxM1*-modified BMSCs reversed the reduction of VE-cadherin protein caused by LPS compared with BMSCs-Vector. Therefore, we believe that FoxM1 may be beneficial for the restoration of vascular integrity.

The Wnt/*β*-catenin pathway contributes to chronic inflammation, organ fibrosis, and asthma [56, 57]. Zhang et al. [58] revealed that upregulation of the Wnt/*β*-catenin pathway attenuated phosgene-induced ALI. Additionally, activating the Wnt/*β*-catenin pathway serves crucial parts in the progression of lung injury and repair during sepsis and ventilator-induced ALI [59]. Moreover, Wnt/*β*-catenin signaling is essential for angiogenesis [60]. Of note, Xie et al. [61] revealed that FoxM1 promoted renal fibrogenesis via activating the Wnt/*β*-catenin pathway. Similarly, Zhang et al. revealed that the increased expression of FoxM1 can promote the nuclear localization of *β*-catenin, thus activating the Wnt/*β*-catenin signaling pathway to regulate the occurrence and development of breast cancer [62]. Additionally, apoptosis was repressed by regulating the FoxM1/Wnt/*β*-catenin pathway [63]. Likewise, Chen et al. [64] found that Wnt-induced deubiquitination of FoxM1 is a novel and important mechanism controlling canonical Wnt signaling and cell proliferation. Furthermore, FoxM1-mediated activation of the Wnt pathway can promote cell proliferation, migration, and epithelial-mesenchymal transition [65]. Here, we discovered that *β*-catenin expression lowered following LPS therapy but was reversed by BMSCs-Vector or BMSCs-FoxM1, and the reversal was more pronounced with BMSCs-FoxM1. Furthermore, our results reveal that a specialized suppressor of the Wnt/*β*-catenin pathway (XAV-939) partially reversed *β*-catenin expression in LPS-induced ALI/ARDS mice following injection of BMSCs-FoxM1. Therefore, we believe that the positive impacts of BMSCs-FoxM1 for the restoration of vascular integrity partially occurred through activating the Wnt/*β*-catenin signaling pathway.

MSCs-based treatments have been widely used in preclinical and clinical studies of various diseases, showing great potential in the treatment of ARDS. However, several therapy-related issues deserve comment. The source of MSCs, the way they are transfused, and their activity in the body can affect the effectiveness of treatment [66, 67]. How to ensure that MSC therapy is safe and efficient under the premise of improving the therapeutic effect is particularly important. Some methods have been employed to improve the value of MSCs in vivo. For example, genetically modified MSCs not only have all the characteristics of stem cells but also can efficiently express foreign genes and enhance their treating effects [68, 69]. Herein, we demonstrated for the first time that FoxM1-modified BMSCs could enhance protection against LPS-induced ALI, which may contribute to the optimization of MSC-based therapies. However, this research has certain restrictions. First, animal models cannot fully recapitulate all the features of human ALI/ARDS, and the efficiency of identical treatments in different manifestations or ALI models requires further investigation. Second, without comorbidities, antibiotic use, or use of ventilators, the LPS-induced ALI/ARDS model cannot fully mimic the pathophysiological process of patients with septic shock. Third, MSCs naturally secrete a variety of paracrine factors, but changes in paracrine factor secretion by MSCs overexpressing FoxM1 and the specific factors that play a therapeutic role in ALI/ARDS development remain to be identified. Such factors may be illuminated by future protein array or microarray analyses.

## 5. Conclusion

In summary, our findings indicate that transplantation of BMSCs overexpressing FoxM1 elicited protective effects against LPS-induced ALI/ARDS by inhibiting inflammation and apoptosis and restoring vascular integrity partially through activating the Wnt/*β*-catenin signaling pathway. These results provide new insight into MSC-based therapeutic strategies for ALI/ARDS.

## Data Availability

The data used to support the findings of this study are included within the article.

## References

[1] Xu Y. P., Zhu J. Q., Feng B. (2021). Immunosuppressive effect of mesenchymal stem cells on lung and gut CD8+ T cells in lipopolysaccharide-induced acute lung injury in mice. *Cell Proliferat*.

[2] Fan E., Brodie D., Slutsky A. S. (2018). Acute respiratory distress syndrome. *Jama-J Am Med Assoc*.

[3] Huppert L. A., Matthay M. A., Ware L. B. (2019). Pathogenesis of acute respiratory distress syndrome. *Semin Resp Crit Care*.

[4] Matthay M. A., Zemans R. L., Zimmerman G. A. (2019). Acute respiratory distress syndrome. *Nature Reviews Disease Primers*.

[5] Curley G. F., Scott J. A., Laffey J. G. (2014). Therapeutic potential and mechanisms of action of mesenchymal stromal cells for acute respiratory distress syndrome. *Current Stem Cell Research & Therapy*.

[6] Cardenes N., Caceres E., Romagnoli M., Rojas M. (2013). Mesenchymal stem cells: a promising therapy for the acute respiratory distress syndrome. *Respiration*.

[7] Gupta N., Su X., Popov B., Lee J. W., Serikov V., Matthay M. A. (2007). Intrapulmonary delivery of bone marrow-derived mesenchymal stem cells improves survival and attenuates endotoxin-induced acute lung injury in mice. *Journal of Immunology*.

[8] Mei S. H. J., McCarter S. D., Deng Y. P., Parker C. H., Liles W. C., Stewart D. J. (2007). Prevention of LPS-induced acute lung injury in mice by mesenchymal stem cells overexpressing angiopoietin 1. *PLoS Medicine*.

[9] Krasnodembskaya A., Samarani G., Song Y. L. (2012). Human mesenchymal stem cells reduce mortality and bacteremia in gram-negative sepsis in mice in part by enhancing the phagocytic activity of blood monocytes. *American Journal of Physiology-Lung Cellular and Molecular Physiology*.

[10] Lee J. W., Krasnodembskaya A., McKenna D. H., Song Y. L., Abbott J., Matthay M. A. (2013). Therapeutic effects of human mesenchymal stem cells in ex vivo human lungs injured with live bacteria. *American Journal of Respiratory and Critical Care Medicine*.

[11] Mei S. H. J., Haitsma J. J., Dos Santos C. C. (2010). Mesenchymal stem cells reduce inflammation while enhancing bacterial clearance and improving survival in sepsis. *American Journal of Respiratory and Critical Care Medicine*.

[12] Walter J., Ware L. B., Matthay M. A. (2014). Mesenchymal stem cells: mechanisms of potential therapeutic benefit in ARDS and sepsis. *The Lancet Respiratory Medicine*.

[13] Nemeth K., Leelahavanichkul A., Yuen P. S. T. (2009). Bone marrow stromal cells attenuate sepsis via prostaglandin E_2_-dependent reprogramming of host macrophages to increase their interleukin-10 production. *Nature Medicine*.

[14] Gupta N., Krasnodembskaya A., Kapetanaki M. (2012). Mesenchymal stem cells enhance survival and bacterial clearance in murine Escherichia coli pneumonia. *Thorax*.

[15] McCarter S. D., Mei S. H. J., Lai P. F. H. (2007). Cell-based angiopoietin-1 gene therapy for acute lung injury. *American Journal of Respiratory and Critical Care Medicine*.

[16] Wu Y. J., Chen L., Scott P. G., Tredget E. E. (2007). Mesenchymal stem cells enhance wound healing through differentiation and angiogenesis. *Stem Cells*.

[17] Lee J. W., Fang X. H., Gupta N., Serikov V., Matthay M. A. (2009). Allogeneic human mesenchymal stem cells for treatment of E. coli endotoxin-induced acute lung injury in the ex vivo perfused human lung. *Proceedings of the National Academy of Sciences*.

[18] Li J. W., Wu X. (2015). Mesenchymal stem cells ameliorate LPS-induced acute lung injury through KGF promoting alveolar fluid clearance of alveolar type II cells. *Eur Rev Med Pharmaco*.

[19] Lu Z. H., Chang W., Meng S. S. (2019). Mesenchymal stem cells induce dendritic cell immune tolerance via paracrine hepatocyte growth factor to alleviate acute lung injury. *Stem Cell Research & Therapy*.

[20] Krasnodembskaya A., Song Y. L., Fang X. H. (2010). Antibacterial effect of human mesenchymal stem cells is mediated in part from secretion of the antimicrobial peptide LL-37. *Stem Cells*.

[21] Lin S., Chen Q., Zhang L. (2021). Overexpression of HOXB4 promotes protection of bone marrow mesenchymal stem cells against lipopolysaccharide-induced acute lung injury partially through the activation of Wnt/*β*-catenin signaling [corrigendum]. *Journal of Inflammation Research*.

[22] Lam E. W. F., Brosens J. J., Gomes A. R., Koo C. Y. (2013). Forkhead box proteins: tuning forks for transcriptional harmony. *Nature Reviews. Cancer*.

[23] Ye H., Kelly T. F., Samadani U. (1997). Hepatocyte nuclear factor 3/fork head homolog 11 is expressed in proliferating epithelial and mesenchymal cells of embryonic and adult tissues. *Molecular and Cellular Biology*.

[24] Zhao Y. D. D., Huang X. J., Yi F. (2014). Endothelial FoxM1 mediates bone marrow progenitor cell-induced vascular repair and resolution of inflammation following inflammatory lung injury. *Stem Cells*.

[25] Ye H. G., Holterman A. X., Yoo K. W., Franks R. R., Costa R. H. (1999). Premature expression of the winged helix transcription factor HFH-11B in regenerating mouse liver accelerates hepatocyte entry into S phase. *Molecular and Cellular Biology*.

[26] Zhang S. W., Teng H. L., Ding Q. L. (2013). FoxM1 involvement in astrocyte proliferation after spinal cord injury in rats. *Journal of Molecular Neuroscience*.

[27] Bolte C., Zhang Y. F., York A. (2012). Postnatal ablation of Foxm1 from cardiomyocytes causes late onset cardiac hypertrophy and fibrosis without exacerbating pressure overload-induced cardiac remodeling. *PLoS One*.

[28] An X. N., Sun X. T., Hou Y. H. (2019). Protective effect of oxytocin on LPS-induced acute lung injury in mice. *Scientific Reports*.

[29] Tang M., Chen L., Li B. (2016). BML-111 attenuates acute lung injury in endotoxemic mice. *The Journal of Surgical Research*.

[30] Feng G., Sun B., Liu H. X., Liu Q. H., Zhao L., Wang T. L. (2019). EphA2 antagonism alleviates LPS-induced acute lung injury via Nrf2/HO-1, TLR4/MyD88 and RhoA/ROCK pathways. *International Immunopharmacology*.

[31] Faller S., Hausler F., Goeft A. (2018). Hydrogen sulfide limits neutrophil transmigration, inflammation, and oxidative burst in lipopolysaccharide-induced acute lung injury. *Scientific Reports*.

[32] Islam D., Huang Y. B., Fanelli V. (2019). Identification and modulation of microenvironment is crucial for effective mesenchymal stromal cell therapy in acute lung injury. *American Journal of Respiratory and Critical Care Medicine*.

[33] Mutlu G. M., Machado-Aranda D., Norton J. E. (2007). Electroporation-mediated gene transfer of the Na+, K+-ATPase rescues endotoxin-induced lung injury. *American Journal of Respiratory and Critical Care Medicine*.

[34] Burnham E. L., Janssen W. J., Riches D. W. H., Moss M., Downey G. P. (2014). The fibroproliferative response in acute respiratory distress syndrome: mechanisms and clinical significance. *European Respiratory Journal*.

[35] Marshall R. P., Bellingan G., Webb S. (2000). Fibroproliferation occurs early in the acute respiratory distress syndrome and impacts on outcome. *American Journal of Respiratory and Critical Care Medicine*.

[36] Wu G. S., Chang F., Fang H. (2021). Non-muscle myosin II knockdown improves survival and therapeutic effects of implanted bone marrow-derived mesenchymal stem cells in lipopolysaccharide-induced acute lung injury. *Annals of Translational Medicine*.

[37] Radeva M. Y., Waschke J. (2018). Mind the gap: mechanisms regulating the endothelial barrier. *Acta Physiologica*.

[38] Ren H., Zhang Q., Wang J., Pan R. (2018). Comparative effects of umbilical cord- and menstrual blood-derived MSCs in repairing acute lung injury. *Stem Cells International*.

[39] Zhang X., Zhang Z., Ju M. (2020). Pretreatment with interleukin 35-engineered mesenchymal stem cells protected against lipopolysaccharide-induced acute lung injury via pulmonary inflammation suppression. *Inflammopharmacology*.

[40] Fang X. H., Neyrinck A. P., Matthay M. A., Lee J. W. (2010). Allogeneic human mesenchymal stem cells restore epithelial protein permeability in cultured human alveolar type ii cells by secretion of angiopoietin-1^∗^, ♦. *The Journal of Biological Chemistry*.

[41] Pati S., Khakoo A. Y., Zhao J. (2011). Human mesenchymal stem cells inhibit vascular permeability by modulating vascular endothelial cadherin/*β*-catenin signaling. *Stem Cells and Development*.

[42] Chen X. X., Wu S. S., Tang L. (2019). Mesenchymal stem cells overexpressing heme oxygenase-1 ameliorate lipopolysaccharide-induced acute lung injury in rats. *Journal of Cellular Physiology*.

[43] Wang C. F., Lv D., Zhang X. B., Ni Z. A., Sun X. F., Zhu C. Q. (2018). Interleukin-10-overexpressing mesenchymal stromal cells induce a series of regulatory effects in the inflammatory system and promote the survival of endotoxin-induced acute lung injury in mice model. *DNA and Cell Biology*.

[44] Zhang S. Q., Jiang W., Ma L. J., Liu Y. H., Zhang X. Y., Wang S. (2018). Nrf2 transfection enhances the efficacy of human amniotic mesenchymal stem cells to repair lung injury induced by lipopolysaccharide. *Journal of Cellular Biochemistry*.

[45] Salerno A., Brady K., Rikkers M. (2020). MMP13 and TIMP1 are functional markers for two different potential modes of action by mesenchymal stem/stromal cells when treating osteoarthritis. *Stem Cells*.

[46] Xu J., Huang Z., Lin L. (2015). miRNA-130b is required for the ERK/FOXM1 pathway activation-mediated protective effects of isosorbide dinitrate against mesenchymal stem cell senescence induced by high glucose. *International Journal of Molecular Medicine*.

[47] Matthay M. A., Zemans R. L. (2011). The acute respiratory distress syndrome: pathogenesis and treatment. *Annual Review of Pathology: Mechanisms of Disease*.

[48] Muller-Redetzky H. C., Suttorp N., Witzenrath M. (2014). Dynamics of pulmonary endothelial barrier function in acute inflammation: mechanisms and therapeutic perspectives. *Cell and Tissue Research*.

[49] Huang X., Dai Z., Cai L. (2016). Endothelial p110*γ*PI3K mediates endothelial regeneration and vascular repair after inflammatory vascular injury. *Circulation*.

[50] Zhao Y. Y., Gao X. P., Zhao Y. D. (2006). Endothelial cell-restricted disruption of FoxM1 impairs endothelial repair following LPS-induced vascular injury. *The Journal of Clinical Investigation*.

[51] Mirza M. K., Sun Y., Zhao Y. D. (2010). FoxM1 regulates re-annealing of endothelial adherens junctions through transcriptional control of beta-catenin expression. *The Journal of Experimental Medicine*.

[52] Huang X. J., Zhao Y. Y. (2012). Transgenic expression of FoxM1 promotes endothelial repair following lung injury induced by polymicrobial sepsis in mice. *PLoS One*.

[53] Minamino T., Komuro I. (2006). Regeneration of the endothelium as a novel therapeutic strategy for acute lung injury. *The Journal of Clinical Investigation*.

[54] Katoh M., Igarashi M., Fukuda H., Nakagama H., Katoh M. (2013). Cancer genetics and genomics of human *FOX* family genes. *Cancer Letters*.

[55] Komarova Y. A., Kruse K., Mehta D., Malik A. B. (2017). Protein interactions at endothelial junctions and signaling mechanisms regulating endothelial permeability. *Circulation Research*.

[56] Katoh M. (2018). Multi-layered prevention and treatment of chronic inflammation, organ fibrosis and cancer associated with canonical WNT/*β*‑catenin signaling activation (review). *International Journal of Molecular Medicine*.

[57] Hussain M., Xu C. Y., Lu M. P., Wu X. L., Tang L. F., Wu X. M. (2017). Wnt/*β*-catenin signaling links embryonic lung development and asthmatic airway remodeling. *Biochimica et Biophysica Acta-Molecular Basis of Disease*.

[58] Zhang L., Zhang F., He D. K., Xu D. J., Zhong Z. Y., Shen J. (2017). Melatonin attenuates phosgene-induced acute lung injury via the upregulation Wnt/beta-catenin pathway. *Int J Clin Exp Patho*.

[59] Villar J., Cabrera N. E., Casula M. (2011). WNT/*β*-catenin signaling is modulated by mechanical ventilation in an experimental model of acute lung injury. *Intensive Care Medicine*.

[60] Liu B., Zhou H., Zhang T. (2021). Loss of endothelial glucocorticoid receptor promotes angiogenesis via upregulation of Wnt/*β*-catenin pathway. *Angiogenesis*.

[61] Xie H., Miao N., Xu D. (2021). FoxM1 promotes Wnt/*β*‐catenin pathway activation and renal fibrosis via transcriptionally regulating multi-Wnts expressions. *Journal of Cellular and Molecular Medicine*.

[62] Zhang J. F., Lu J. Y., Chen Y., Li H., Lin L. S. (2020). WHSC1 promotes wnt/*β*-catenin signaling in a FoxM1-dependent manner facilitating proliferation, invasion and epithelial-mesenchymal transition in breast cancer. *Journal of Receptors and Signal Transduction*.

[63] Shi C. J., Zhang H., Wang M. (2022). OPA interacting protein 5 antisense RNA 1 expedites cell migration and invasion through FOXM1/ Wnt/*β*-catenin pathway in pancreatic cancer. *Digestive Diseases and Sciences*.

[64] Chen Y. H., Li Y., Xue J. F. (2016). Wnt-induced deubiquitination FoxM1 ensures nucleus *β*‐catenin transactivation. *The EMBO Journal*.

[65] Zhang H., Zhou Q. Q., Shen W. M. (2022). Circ-FOXM1 promotes the proliferation, migration and EMT process of osteosarcoma cells through FOXM1-mediated Wnt pathway activation. *Journal of Orthopaedic Surgery and Research*.

[66] Wang L., Feng Y., Dou M. (2022). Study of mesenchymal stem cells derived from lung-resident, bone marrow and chorion for treatment of LPS-induced acute lung injury. *Respiratory Physiology & Neurobiology*.

[67] Matthay M. A., Calfee C. S., Zhuo H. J. (2019). Treatment with allogeneic mesenchymal stromal cells for moderate to severe acute respiratory distress syndrome (START study): a randomised phase 2a safety trial. *The Lancet Respiratory Medicine*.

[68] Li Z., Song Y. Q., Yuan P. S. (2020). Antibacterial fusion protein BPI21/LL-37 modification enhances the therapeutic efficacy of hUC-MSCs in sepsis. *Molecular Therapy*.

[69] Desterke C., Griscelli F., Imeri J. (2021). Molecular investigation of adequate sources of mesenchymal stem cells for cell therapy of COVID-19-associated organ failure. *Stem Cells Translational Medicine*.

